# Challenges in the Management of SARS-CoV2 Infection: The Role of Oral Bacteriotherapy as Complementary Therapeutic Strategy to Avoid the Progression of COVID-19

**DOI:** 10.3389/fmed.2020.00389

**Published:** 2020-07-07

**Authors:** Gabriella d'Ettorre, Giancarlo Ceccarelli, Massimiliano Marazzato, Giuseppe Campagna, Claudia Pinacchio, Francesco Alessandri, Franco Ruberto, Giacomo Rossi, Luigi Celani, Carolina Scagnolari, Cristina Mastropietro, Vito Trinchieri, Gregorio Egidio Recchia, Vera Mauro, Guido Antonelli, Francesco Pugliese, Claudio Maria Mastroianni

**Affiliations:** ^1^Department of Public Health and Infectious Diseases, Sapienza University of Rome, Rome, Italy; ^2^Department of Clinical and Molecular Medicine, Sapienza University of Rome, Rome, Italy; ^3^Department of Anesthesia and Intensive Care Medicine, Sapienza University of Rome, Rome, Italy; ^4^School of Biosciences, Veterinary Medicine University of Camerino, Camerino, Italy; ^5^Laboratory of Virology, Department of Molecular Medicine, Affiliated to Istituto Pasteur Italia - Cenci Bolognetti Foundation, Sapienza University, Rome, Italy

**Keywords:** COVID-19, SARS-CoV-2, bacteriotherapy, probiotic, lactobacillus, gut-lung axis, gut

## Abstract

**Background:** Gastrointestinal disorders are frequent in COVID-19 and SARS-CoV-2 has been hypothesized to impact on host microbial flora and gut inflammation, infecting intestinal epithelial cells. Since there are currently no coded therapies or guidelines for treatment of COVID-19, this study aimed to evaluate the possible role of a specific oral bacteriotherapy as complementary therapeutic strategy to avoid the progression of COVID-19.

**Methods:** We provide a report of 70 patients positive for COVID-19, hospitalized between March 9th and April 4th, 2020. All the patients had fever, required non-invasive oxygen therapy and presented a CT lung involvement on imaging more than 50%. Forty-two patients received hydroxychloroquine, antibiotics, and tocilizumab, alone or in combination. A second group of 28 subjects received the same therapy added with oral bacteriotherapy, using a multistrain formulation.

**Results:** The two cohorts of patients were comparable for age, sex, laboratory values, concomitant pathologies, and the modality of oxygen support. Within 72 h, nearly all patients treated with bacteriotherapy showed remission of diarrhea and other symptoms as compared to less than half of the not supplemented group. The estimated risk of developing respiratory failure was eight-fold lower in patients receiving oral bacteriotherapy. Both the prevalence of patients transferred to ICU and mortality were higher among the patients not treated with oral bacteriotherapy.

**Conclusions:** A specific bacterial formulation showed a significant ameliorating impact on the clinical conditions of patients positive for SARS-CoV-2 infection. These results also stress the importance of the gut-lung axis in controlling the COVID-19 disease.

## Introduction

Understanding the invasive process of SARS-COV-2 is essential. We know that the entry points for the virus into the body, such as ACE2 receptors, are enzymes that are linked to intestinal cells. Coronaviruses constantly change their binding patterns as they evolve, and the potential target in the lungs also varies, but not in the small intestine, where it remains constant. The cells of the intestinal mucosa (enterocytes) could, therefore, be a reservoir for coronaviruses (1). In the acute phase, only 10% of coronavirus disease 19 (COVID-19) patients present virus cDNA in the blood, but almost 50% of them excrete it in the stools. The infectious form of the virus was even identified several times, suggesting that the orofecal route is a mode of contamination (1). The gut involvement might explain the wide variation in viral load from one test to another in the same person as if the virus were hiding there (2). Chinese researchers have investigated changes in the microbiota in the patients who have died for COVID-19 infection. The sequencing of their microbiota revealed a significant decrease in *bifidobacteria* and *lactobacilli*, the main families of symbiotic bacteria, as well as an increase in opportunistic bacteria such as *Corynebacterium* or *Ruthenibacterium* (1). Intestinal dysbiosis has a long-reaching immune impact on the pulmonary immune system (3), and hence might be an additional risk for respiratory distress induced by COVID-19. In this context, the use of oral bacteriotherapy might be an option. Some strains of *lactobacilli* and *bifidobacteria* have a protective role against influenza virus, rhinovirus, respiratory syncytial virus, adenovirus, and pneumovirus (4, 5). We report here our observation on patients supplemented with oral bacteriotherapy in addition to the current anti-COVID-19 treatment (hydroxychloroquine, azithromycin, tocilizumab). The comparison group was COVID-19 positive subjects not treated with oral bacteriotherapy, hospitalized in the same clinic at the same time. Our results stress the importance of the gut-lung axis in the control of the COVID-19 illness (6, 7).

## Study Population, Settings and Data Collection

The patients evaluated in this study were hospitalized at the Department of Infectious Diseases, Policlinico Umberto I, “Sapienza” University of Rome, Italy, between March 9th, 2020 and April 4th, 2020 (sub-intensive care unit for COVID-19). Ethical approval was obtained from Ethics Committee of Policlinico Umberto I (approval number/ID Prot. 109/20209). All the patients were staying at home before their referral to the Emergency Department, and from there to our Department. Oropharyngeal and nasopharyngeal swabs for diagnosis of COVID-19 were performed in duplicate for SARS-CoV-2 E and S gene by a reverse transcriptase polymerase chain reaction (RT-PCR). All the patients were positive for COVID-19 and met the following clinical criteria: Fever: > 37.5°C, need of non-invasive oxygen therapy, and CT lung involvement on imaging more than 50%. They were diagnosed with symptomatic COVID-19 disease state, which, however, at the time of evaluation did not require endotracheal intubation and invasive mechanical ventilation. Oxygen therapy was delivered via Venturi mask in spontaneous breathing patients; if hypoxemia persisted continuous positive airway pressure (CPAP) was applied. Dyspnea was defined as “a subjective experience of breathing discomfort that consists of qualitatively distinct sensations that vary in intensity” (8). Acute diarrhea was defined as a stool with increased water content, volume, or frequency that lasts <14 days (9). High-resolution CT scan was used to identify lung involvement according to the official diagnosis and treatment protocol (6th edition) declared by the National Health Commission of China. Typical CT findings of COVID-19 are (1) ground-glass opacities, (2) consolidation, (3) reticular pattern, (4) crazy paving pattern (10). Patients with severe acute hypoxemia due to COVID-19 pneumonia and in need for invasive mechanical ventilation were referred to the Intensive Care Unit (ICU) of Policlinico Umberto I. Since there are, currently, no coded therapies or guidelines for the medical treatment of COVID-19, the patients were treated with hydroxychloroquine (HCQ) 200 mg bid, antibiotics (ABX) (azithromycin 500 mg) and Tocilizumab (TCZ) dosage is 8 mg/kg (up to a maximum of 800 mg per dose) with an interval of 12 h for two times, eventually plus oxygen. In addition to the above treatments, randomly chosen patients initiated oral bacteriotherapy on March 13th. For each patient, the Charlson comorbidity index (11), the oxygen-support requirement, as well as, laboratory values comprising alanine aminotransferase (ALT), aspartate aminotransferase (ALT), hemoglobin (Hb), pH, hydrogen carbonate (${\text{HCO}}_{3}^{-}$), Lactic acid and arterial carbon anhydride pressure (PaCO_2_) were determined at baseline. The observed partial pressure of arterial oxygen (PaO_2_), the fraction of inspired oxygen FiO_2_, the disappearance of symptoms associated to COVID-19, adverse events, and the number of patients transferred to ICU were collected at 24 h, 48 h, 72 h, and 7 days from the start of oral bacteriotherapy and hospitalization for all the patients independently from the treatments. Patients were considered positive for respiratory failure when the determined PaO_2_/FiO_2_ ratio was <300. Since this is a retrospective real-life emergency data collection, some laboratory data were unavailable. In particular, in the case of significant clinical and respiratory gas exchange improvement, sometimes the clinician has not repeated the follow up blood gas analysis, considering it an unnecessary painful invasive procedure.

### Oral Bacteriotherapy

The formulation administered in this study contained: *Streptococcus thermophilus* DSM 32345*, L.acidophilus* DSM 32241, *L. helveticus* DSM 32242*, L. paracasei* DSM 32243, *L. plantarum* DSM 32244, *L. brevis* DSM 27961, *B. lactis* DSM 32246, *B. lactis* DSM 32247. Ormendes SA, Lausanne, Switzerland which gifted the product Sivomixx® (SivoBiome® in USA) is responsible for the standardization of the product in terms of enzymatic content, biochemical and immunological profile. The oral bacteriotherapy involved the use of 2,400 billion bacteria per day. The formulation was administered in three equal doses per day.

### Statistical Analysis

No sample-size calculations were performed. The categorical variables were compared using the χ^2^ test and showed as absolute frequencies and percentage. The Shapiro–Wilk test was used to test the normality of distribution of continuous variables. When they were not normally distributed, logarithmic transformation was performed in accordance to BoxCox transformation with −0.25 ≤ λ ≤ 0.25. For normally distributed continuous variables, mean values between two groups were compared by Student's *t*-test and showed as mean ± SD (Standard Deviation); for data not normally distributed, the Mann–Whitney test was used and indicated as median (25th−75th). The longitudinal analysis of data relative to respiratory failure in relation to the “not treated vs. treated” group was performed by a General Linear Mixed Model with the GLIMMIX procedure considering the binary as distribution and logit as link function. The Benjamini–Hochberg False Discovery Rate (FDR) correction was used to account for multiple hypothesis testing when necessary. A *p* < 0.05 was considered statistically significant. All statistical analyses were performed by using SAS v.9.4 and JMP v. 14 (SAS Institute Inc., Cary, NC, USA).

## Results

Data relative to 70 subjects positive to the SARS-CoV-2 test (median age, 59 years, interquartile range, 50–70) were collected during period March 9th–April 4th, 2020. None of the patients had recently traveled to China or South Korea or Iran. Patients were unable to confirm if they had contact with persons infected with COVID-19 and were also not accurate in recalling the exact duration of the symptoms before hospital admission. The proportion of females (29, 41.4%) was lower respect to the percentage of males (41, 58.6%). Symptomatology of patients at admission was: fever (66, 94.3%), cough (54, 77.1%), dyspnea (44, 62.9%), headache (11, 15.7%), asthenia (15, 21.4%), myalgia (4, 5.7%), diarrhea (33, 47.1%), while 56 (80.0%), presented comorbidities in a range of 1 to 6. A group of 28 subjects received Oral Bacteriotherapy (OB+), while another group of 42 individuals not supplemented with oral bacteriotherapy (OB–) was the comparison group. Table 1 shows the characteristics of patients at admittance. No statistically significant differences were observed between the OB+ group and the OB– one respect to sex, age, AST, ALT, Hb, Body Mass Index (BMI), and Charlson comorbidity index at baseline. No significant differences between groups were also found for respiratory parameters as well as for the proportion of subjects presenting diarrhea, fever, cough, dyspnea, headache, asthenia, and myalgia. Furthermore, all patients had clinical and radiological signs compatible with COVID-19 pneumonia and needed respiratory assistance in the hospital setting but not resuscitation support. The two groups of patients were homogeneous respect to the proportion of subjects needing non-invasive oxygen support delivered via Venturi mask in spontaneous breathing or by continuous positive airway pressure (CPAP).

**Table 1:** Characteristics of the groups of patients obtained on the base of bacteriotherapy administration.

	**OB+**		**OB–**		
	**(*****N*** **=** **28)**		**(*****N*** **=** **42)**		
**Characteristics**	**Obs**.	**Values**	**Obs**.	**Values**	***p*-value**
Age^*^ (year)	28	59 ± 14.4	42	60.5 ± 14.2	0.66
Gender male	28	17 (60.7)	42	24 (57.1)	0.77
BMI^*^ (kg/m^2^)	27	24.7 ± 3.4	42	23.4 ± 3.5	0.13
ALT^*^(IU per liter)	27	22.7 ± 10.5	42	30.5 ± 22.2	0.16
AST^*^(IU per liter)	27	37 ± 33.5	42	40.5 ± 33	0.56
Hb (g/dL)	27	13 (12–14)	42	13 (12–14)	0.58
Charlson index	28	2 (1–3)	41	2 (1–3)	0.64
Symptoms					
Fever	28	27 (96.4)	42	39 (92.9)	0.53
Cough	28	22 (78.6)	42	32 (76.2)	0.82
Dyspnea	28	20 (71.4)	42	24 (57.1)	0.23
Asthenia	28	6 (21.4)	42	9 (21.4)	1.0
Headache	28	3 (10.7)	42	8 (19.0)	0.35
Myalgia	28	2 (7.1)	42	2 (4.8)	0.67
Diarrhea	28	14 (50.0)	42	19 (45.2)	0.70
Respiratory parameters					
FiO_2_ (%)	27	31 (21–43)	39	21 (21–35)	0.17
SO_2_ (%)	24	99 (97–99)	36	99 (97–100)	0.89
pH	26	7.5 (7.4–7.5)	35	7.5 (7.4–7.5)	0.71
${\text{HCO}}_{3}^{-}$ (mmol/L)	26	25.47 ± 3.37	33	25.58 ± 2.74	0.89
Lactic acid^*^ (mmol/L)	26	0.9 ± 0.4	34	0.9 ± 0.3	0.74
pO_2_ (mmHg)	27	89 (76–103)	39	86 (77–97)	0.73
pCO_2_ (mmHg)	26	36 (32–39)	36	36 (34–39)	0.40
Oxygen support					
Venturi mask	28	25 (89.3)	42	38 (90.5)	0.87
CPAP	28	3 (10.7)	42	3 (7.1)	0.60

*The p-values are relative to the chi-square test and Mann–Whitney and when less of 0.05 these comparisons showed no significant differences. Obs., number of available observations for each group*.

* *data log_10_-transformed. OB– (oral bacteriotherapy not administered group), OB+ (oral bacteriotherapy administered group)*.

Notably, at admittance, a significantly higher proportion of patients with respiratory failure was present in the group treated with oral bacteriotherapy respect to the OB– one (OB– 11/42, 26.2%; OB+ 14/28, 50%; *p* = 0.042). Therefore, all enrolled patients were classifiable in stage III (Severe pneumonia–Severe COVID-19) of the syndromic classification proposed by the Italian Society of Anesthesia and Resuscitation (SIAARTI) (12).

For what concerns drug therapies, both groups did not differ for number, type, and combinations of administered drugs during the period of hospitalization (Table 2). The median time from diagnosis to the start of oral bacteriotherapy administration was 1 day (min 0–max 2) and duration of treatment was 14 days for all patients.

**Table 2:** Drug therapies administered to the groups of subjects as determined by the administration of bacteriotherapy.

	**OB+**		**OB–**		
	**(*****N*** **=** **28)**		**(*****N*** **=** **42)**		
	**Obs**.	**Values**	**Obs**.	**Values**	***p*-value**
Administered drugs–no. (%)
HCQ	28	25 (89.3)	42	40 (95.2)	0.34
TCZ	28	7 (25)	42	16 (38.1)	0.25
ABX	28	11 (39.3)	42	21 (50)	0.38
Administered drugs–no. (%)					0.16
None	28	2 (7.1)	42	1 (2.4)	
One drug	28	13 (46.4)	42	11 (26.2)	
Two drugs	28	9 (32.1)	42	24 (57.1)	
Three drugs	28	4 (14.3)	42	6 (14.3)	
Combinations of drugs–no. (%)					
HCQ/TCZ/ABX	28	4 (14.3)	42	6 (14.3)	1.0
HCQ/TCZ	28	2 (7.1)	42	9 (21.4)	0.11
HCQ/ABX	28	6 (21.4)	42	15 (35.7)	0.20
STCZ/ABX	28	1 (3.6)	42	0 (0)	0.22

*A p ≤ 0.05 was considered statistically significant. ABX, antibiotics; HCQ, hydroxychloroquine; IRQ, interquartile range; Obs., number of available observations for each group; TCZ, Tocilizumab*.

**Figure 1:**
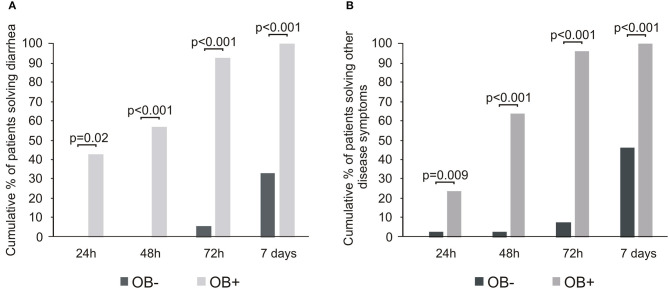
Color-coded barplots based on probiotic administration showing the disappearance of diarrhea **(A)** as well as other symptoms **(B)** at different time points. The Benjamini Hochberg FDR correction was used to account for multiple hypothesis testing. Statistical significance between the group at alpha level 0.05 was also reported.

**Figure 2:**
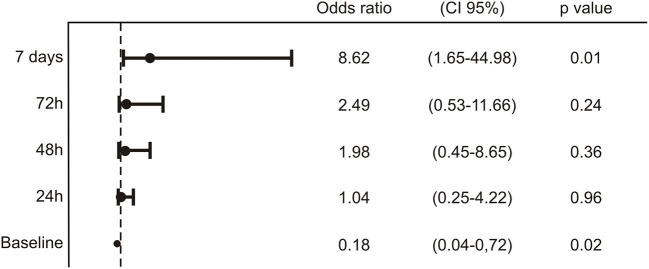
Analysis of the longitudinal data for the respiration variable in relation to the “OB– vs. OB+” group performed by GLIMMIX. For each time point, the odds ratio, the confidence interval 95% and the statistical significance were reported.

The oral bacterial administration was associated with the disappearance of diarrhea in all the patients within 7 days. Interestingly, a large proportion of OB+ subjects (6/14, 42.9%) solved diarrhea within 24 h and almost the totality (13/14, 92.9%) within 3 days (Figure 1A). Also, the other signs and symptoms–fever, asthenia, headache, myalgia, and dyspnea–considered cumulatively, presented a similar trend, more evident from the second day of bacteriotherapy (Figure 1B). Notably, less than half of patients not treated with bacteriotherapy experienced the disappearance of diarrhea or other symptoms within 7 days. For what concerns the respiratory outcome, by applying the General Linear Mixed model with the GLIMMIX procedure, we observed a significant difference in the evolution of respiratory outcome between the OB+ group and the OB– one (*p* < 0.001). After 7 days of treatment, the calculated model showed an 8-fold significantly decreased risk to evolve a respiratory failure, with the need of resuscitation support i.e., in need for prone ventilation or extracorporeal membrane oxygenation (ECMO) for patients administered with bacteriotherapy respect to the OB– individuals (Figure 2).

QT interval prolongation, hepatic and renal abnormalities, and immunosuppression were monitored carefully, also for the propensity of the QT interval to increase in patients treated with azithromycin. No adverse events were recorded. Patients treated with Tocilizumab reported a sense of asthenia after the administration of the drug and a reduction of the blood pressure, which did not require any medical treatment. As of April 4th, 2020, although not statistically significant, the OB– group showed a higher prevalence of patients transferred to the ICU for mechanical ventilation (OB– vs. OB+; 2/42, 4.8% vs. 0/28, 0.0%) or coming to a lethal outcome (OB– vs. OB+; 4/42, 9.5% vs. 0/28, 0.0%). The observed prevalence of patients with lethal outcome within the control group was in line with that (mean ± SD 9.4 ± 1.7%;) recorded in Italy in the period March 9th–April 4th, 2020 (13). All the patients also treated with bacteriotherapy survived the COVID-19 illness, and none required invasive mechanical ventilation and ICU admission.

## Discussion

This report comes from doctors in the “trenches” during the Italian war against the COVID-19 infection. The urgency of the COVID-19 pandemic shifted the balance between waiting for evidence before deciding whether to administer therapy or creating evidence during routine patient care, in favor of the second choice. The United States Food and Drug Administration (FDA) has permitted an emergency-use authorization to prescribe the hydroxychloroquine (14). The WHO, CDC, and FDA have not taken a position on the use of Tocilizumab in COVID-19, even though China's National Health Commission recommends it for use in COVID-19 patients but only if elevated IL-6 levels are present (15). Also, Italian clinicians are utilizing a variety of empirical approaches to managing COVID-19, in a “learn while doing” method (16). There is an urgent need to determine which interventions against COVID-19 are the best, but in the absence of clinical trials to guide the management, not collecting the data from the use of off-label therapies it is a missed opportunity. Here we report a “snapshot” on 70 patients hospitalized at the Department of Infectious Diseases between March 9th and April 4th, 2020. A group of patients has been treated with hydroxychloroquine, Tocilizumab, and antibiotics alone or in combination, while, a second group of subjects were administered with oral bacteriotherapy in addition to the standard drug therapy. Results evidenced a worse survival, as well as, a higher risk of transfer to an intensive resuscitation for the patient not supplemented with bacteriotherapy respect to the supplemented one. Also, the estimated risk to develop respiratory failure during COVID-19 course was more than eight times lower in the group treated with oral bacteriotherapy respect to the not treated one. As for the other signs and symptoms associated with COVID-19, i.e., diarrhea, fever, cough, dyspnea, asthenia, myalgia a significant improvement is already evident as early as after 24–48 h after the start of the bacteriotherapy. There are potential anatomical communications and complex pathways involving the gut-lung axis (GLA) (5). The mesenteric lymphatic system is the pathway between the lungs and the intestine, through which intact bacteria, their fragments or metabolites can cross the intestinal barrier to reach systemic circulation and influence the pulmonary immune response (17–19). Intestinal metabolites significantly affect not only local intestinal immunity but also other organs through the lymphatic and circulatory system. For example, short chain fatty acids (SCFA) produced primarily by bacterial fermentation of dietary fiber, act in the lungs as signaling to attenuate inflammatory and allergic responses (20, 21). Mice with SCFA receptor deficiency show increased inflammatory responses in experimental models of asthma (19). Human cells possess antioxidative defense systems for their protection against reactive oxygen species (ROS) generated by viruses; however, viral infections often inhibit such a response (22). There is no reason to believe that that this is not true also for COVID-19 (23). We hypothesized that in patients infected by COVID-19, a bacterial formulation with the “appropriate” biochemical and immunological profile might trigger several protective biological functions. The bacterial strains present in the product we administered enhance the production of both the *nuclear factor erythroid 2p45-related factor 2* (Nrf2) and its target *Heme oxygenase-1* (HO-1) (24). These molecules exert antiviral activity through a reduction of oxidative stress. Nrf2 and HO-1 have significant antiviral activity against a wide variety of viruses, including Human immunodeficiency virus (HIV), influenza virus, respiratory syncytial virus, dengue virus, and Ebola virus among others (25–29). Notably, beneficial properties of HO-1 expression have been reported for viruses that produce lung disease. Mice that overexpress HO-1 in the lungs display less inflammatory cell infiltration into the lungs and decreased apoptosis of respiratory epithelial cells, as compared to control mice. Therefore, HO-1 expression prevents an exacerbated immune response in this tissue and subsequent damage (26). The collection of clinical data, examination, and nursing of COVID-19 patients is challenging for the risk of virus transmission. The COVID-19 is present in the stools, even in discharged patients, with potential recurrence and transmission of the virus (30, 31). Our initiative aimed to modulate the gut-lung axis, facilitate patient management and possibly determine the outcome of lung infection. Oral bacteriotherapy has shown a statistically significant impact on the clinical conditions of COVID-19 patients. Having considered the different outcomes and unethical to deprive a percentage of COVID-19 patients of the chance to get oral bacteriotherapy, we did not include more patients or extended the time of observation. Pending the results of confirmatory clinical trials, this report is aimed at providing an interim suggestion for improving the management of the COVID-19 illness, keeping in mind that different bacterial preparations may have quite different outcomes (32).

## Data Availability Statement

All datasets presented in this study are included in the article/supplementary material.

## Ethics Statement

The studies involving human participants were reviewed and approved by Ethics Committee of Policlinico Umberto I. The patients/participants provided their written informed consent to participate in this study.

## Author Contributions

Gd'E, GCe, and MM contributed substantially to the conception and design of the study and interpretation and wrote the manuscript. GCa, CP, FA, FR, GR, LC, CS, CM, VT, GER, and VM contributed substantially to the the acquisition of data and the analysis. GA, FP, and CMM drafted or provided critical revision of the article and provided final approval of the version to publish. GR contributed substantially to data interpretation.

## Conflict of Interest

The authors declare that the research was conducted in the absence of any commercial or financial relationships that could be construed as a potential conflict of interest.

## Abbreviations

ABX: antibiotics
ALT: alanine aminotransferase
ALT: aspartate aminotransferase
CI: confidence interval
COVID-19: coronavIrus disease 19
CPAP: continuous positive airway pressure
ECMO: extracorporeal membrane oxygenation
FDR: False Discovery Rate
GLA: gut lung axis
Hb: hemoglobin
HCQ: hydroxychloroquine
HIV: Human immunodeficiency virus
HO-1: Heme oxygenase-1
ICU: Intensive Care Unit
IRQ: interquartile range
Nrf2: nuclear factor erythroid 2p45-related factor 2
OB–: oral bacteriotherapy not administered group
OB+: oral bacteriotherapy administered group
ROS: reactive oxygen species
SCFA: short chain fatty acids
TCZ: Tocilizumab.
